# Load sharing between synergistic muscles characterized by a ligand-binding approach and elastography

**DOI:** 10.1038/s41598-023-45037-y

**Published:** 2023-10-25

**Authors:** Gustavo A. Grinspan, Liliam Fernandes de Oliveira, Maria Clara Brandao, Andrés Pomi, Nicolás Benech

**Affiliations:** 1 Sección Biofísica y Biología de Sistemas, Facultad de Ciencias, Universidad de la República, Iguá 4225, 11400 Montevideo, Uruguay; 2 Laboratorio de Acústica Ultrasonora, Facultad de Ciencias, Universidad de la República, Iguá 4225, 11400 Montevideo, Uruguay; 3 Laboratório de Análise do Movimento e Fisiologia do Exercício, Programa de Engenharia Biomédica, Universidade Federal do Rio de Janeiro, Av. Horácio Macedo 2030, Rio de Janeiro, 21941-590 Brazil

**Keywords:** Bone quality and biomechanics, Muscle contraction, Acoustics

## Abstract

The skeletal muscle contraction is determined by cross-bridge formation between the myosin heads and the actin active sites. When the muscle contracts, it shortens, increasing its longitudinal shear elastic modulus (${\mu }_{L}$). Structurally, skeletal muscle can be considered analogous to the molecular receptors that form receptor–ligand complexes and exhibit specific ligand-binding dynamics. In this context, this work aims to apply elastography and the ligand-binding framework to approach the possible intrinsic mechanisms behind muscle synergism. Based on the short-range stiffness principle and the acoustic–elasticity theory, we define the coefficient $C$, which is directly related to the fraction saturation of molecular receptors and links the relative longitudinal deformation of the muscle to its ${\mu }_{L}$. We show that such a coefficient can be obtained directly from ${\mu }_{L}$ estimates, thus calculating it for the biceps brachii, brachioradialis, and brachialis muscles during isometric elbow flexion torque (τ) ramps. The resulting $C\left(\tau \right)$ curves were analyzed by conventional characterization methods of receptor–ligand systems to study the dynamical behavior of each muscle. The results showed that, depending on muscle, $C\left(\tau \right)$ exhibits typical ligand-binding dynamics during joint torque production. Therefore, the above indicates that these different behaviors describe the longitudinal shortening pattern of each muscle during load sharing. As a plausible interpretation, we suggested that this could be related to the binding kinetics of the cross-bridges during their synergistic action as torque increases. Likewise, it shows that elastography could be useful to assess contractile processes at different scales related to the change in the mechanical properties of skeletal muscle.

## Introduction

Elastography has become a widely accepted methodology to assess the mechanical properties of soft tissues. This methodology has been initially described as a complementary tool for the medical diagnosis of pathologies such as liver fibrosis and breast tumors^1–4^. However, in recent years, it has also begun to be used in biomechanics to assess the shear elastic modulus of skeletal muscle ($\mu$). In this sense, shear wave (SW) elastography methods, such as transient elastography (TE) and supersonic shear imaging (SSI), are the gold standard methods to assess muscle elasticity in-vivo and non-invasively^5,6^. By combining high-frequency ultrasonic waves (10^6^ Hz) with low-frequency waves (100–1000 Hz), these methods exhibit high spatial resolution (< 1 mm) and good contrast in the characterization of shear elastic modulus.

Among the most relevant applications of SW elastography in biomechanical research, several works addressed the relationship between shear elasticity and different muscular variables. Thus, some authors assessed the torque vs. elasticity relationship in different muscles and load conditions^7–10^. Other works have aimed to expand on this research by examining the change of elasticity regarding the electromyographic (EMG) activity and joint torque level^11,12^. Also, other studies have sought to correlate the change in muscle elasticity with functional aspects. For example, SW elastography has been used to assess regional differences in the shear elastic modulus of the elbow flexor muscles after eccentric exercise and its relationship to muscle length^13^. Likewise, there have been studies to relate the changes in muscle elasticity with load sharing and force production in synergistic muscles, both in normal and fatiguing conditions^14–17^.

Skeletal muscle contraction is the process that underlies all the behaviors characterized in the previously referred works. According to the intrinsic property of short-range stiffness (SRS), the skeletal muscle undergoing isometric contractions is characterized by force-stiffness properties^18–21^. This follows directly from the cross-bridge muscle model, where an increase in bound actin-myosin cross-bridges increases Young’s modulus and the shear elastic modulus of the muscle^22^. On the other hand, acousto-elasticity theory links the shear wave speed to the uniaxial stress^23,24^. Recently, it has been shown that this theory can be adapted to study shear wave propagation in a homogeneous, transversely isotropic incompressible solid, subject to uniaxial stress, as the skeletal muscle during isometric contraction^25^. Thus, the SRS and the acoustoelasticity theory can potentially link the shear wave propagation to the contractile processes underlying the geometric and mechanical changes typically associated with skeletal muscle contraction.

As pointed out in classic papers from the 1960s, the structural basis of the longitudinal deformation that characterizes muscle contraction depends on the overlap between actin and myosin filaments through the formation of cross-bridges^26–28^. In this regard, it is important to highlight the structural and functional analogy between cross-bridge formation and the ligand-binding behaviors exhibited by the molecular receptors. The main feature of these molecules is that they have one or more specific sites to bind their ligands, becoming a receptor-ligand complex when saturated. The ligands are the molecules that allow the receptors to perform their biological function when attached to the binding site, which increases the fraction saturation between the occupied sites and the total sites. Depending on the receptor type, this association may describe cooperative or hyperbolic binding dynamics^29^. Similarly, when skeletal muscle contracts, it develops force through the formation of cross-bridges, by increasing the fraction of the myosin heads attached to the actin active sites. Likewise, several works have shown that the formation of actin-myosin cross-bridges follows the same binding dynamics as the molecular receptors, depending on certain physiological factors closely related to muscle contraction^30–40^.

In this context, since shortening and the related changes in muscle properties during its isometric contraction depend on cross-bridge formation, this system is suitable to be analyzed by the receptor-ligand framework. However, the biomechanical studies driven by elastography have not yet addressed the link between muscle elasticity, force generation, and the binding dynamics among the myosin heads and the active sites of the actin filaments. We believe that elastography can be a valuable tool to characterize the contractile processes that underlie the specific muscle functions at the macroscopic scale. In this regard, the present work aims to apply elastography and the ligand-binding framework to account for the dynamic changes in the longitudinal deformation of muscle during the isometric contraction of the elbow flexors. Thus, it provides a new elastography-driven approach to understanding the possible underlying basis behind load sharing between synergistic muscles.

## Materials and methods

### Subjects

Thirteen healthy male volunteers participated in the study (age 27.92 ± 6.90 year, height 179.85 ± 3.31 cm, weight 84.69 ± 12.70 kg). They were informed about the methods, procedures, and the purpose of the study. All participants provided their written informed consent. The experimental design of the study was conducted according to the last version of the Helsinki statement and was approved by the Ethical Committee of the Faculty of Medicine (UdelaR, Uruguay, File No. 071140-001398-11).

### Instrumentation

#### Ergometry

**Figure 1:**
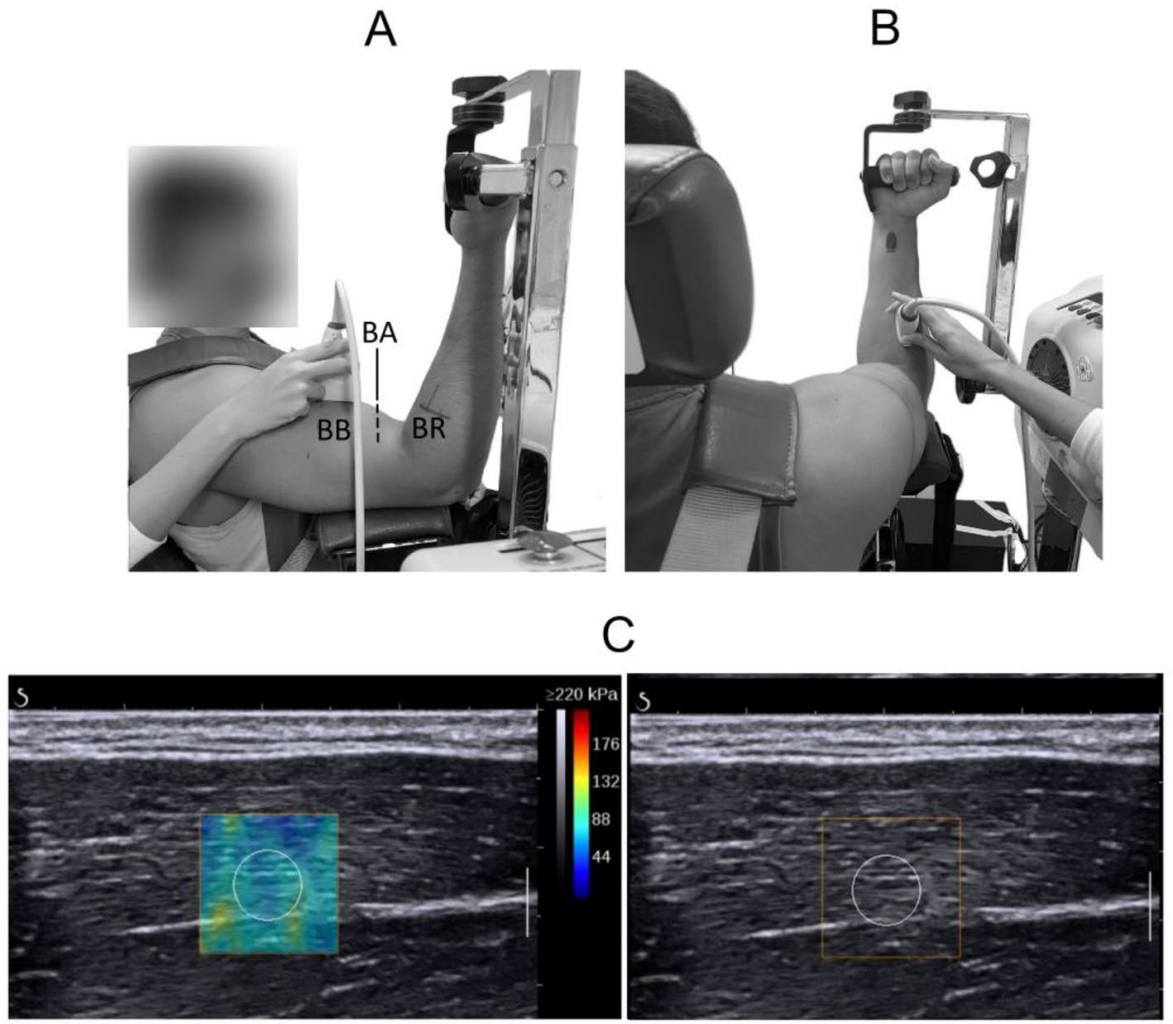
Examples of ${\mu }_{L}$ measurements. (**A,B**) Placement of the ultrasound transducer over the free surface of the biceps brachii (BB) and brachioradialis (BR) muscles, respectively. (**C**) Echographic image showing the ROI chosen for the measurements. At the right, the echographic image without the color map is shown. The colored region in the image at the left depicts the shear elasticity map according to the corresponding color scale.

A research isokinetic dynamometer (Biodex System 4; Biodex Medical, Shirley, NY) was used to measure the angle and torque production of the elbow joint. During the data collection, the volunteers were positioned with their right shoulder and elbow flexed at 90° and the forearm supinated. The elbow joint was aligned coaxially with the axis of the dynamometer (Fig. 1).

#### Elastography

An Aixplorer ultrasonic scanner (Supersonic Imagine, Aix en Provence, France) with a linear transducer array (2–10 MHz, SuperLinear 10-2, Vermon, Tours, France) was used in SSI mode to obtain the shear elasticity map of the tissue. Briefly, this method creates a bulk quasi-plane wave inside the medium using the acoustic radiation force. These waves are excited using the Mach cone technique, which successively induces an ultrasound beam focus (i.e., pushing beam) at different depths. The main characteristic of a Mach cone is the source displacement velocity, which moves faster than the wave, thus generating a quasi-plane wave through constructive interferences. Then, ultrafast echographic imaging sequences are performed to acquire successive radio-frequency data at a high frame rate. A speckle tracking algorithm was used to retrieve the displacement field and calculate the shear wave velocity (${V}_{s}$). A more detailed description of this method can be found in Tanter et al.^41^ and Bercoff et al.^5^.

The measurements were made in the biceps brachii (BB), brachioradialis (BR), and brachialis (BA) muscles, placing the echographic probe aligned regarding their shortening direction (${x}_{3}$). Considering the architecture of such muscles as a set of fibers arranged preferentially along the ${x}_{3}$, and orthogonally regarding the ${x}_{1}$ and ${x}_{2}$ directions, the hypothesis of a transversely isotropic material is the most reasonable to model them. This model considers the muscle as a soft solid with one preferred direction regarding the fiber arrangement. It can be described by the five independent constants ${\mu }_{L}$, ${E}_{L}$, ${E}_{T}$, ${\nu }_{LT}$, and ${\nu }_{LT}$. Here, ${\mu }_{L}$ is the shear elastic modulus relative to deformations along the fibers, and ${E}_{L}$ and ${E}_{T}$ are the Young’s moduli along and transverse to the fibers, respectively. ${\nu }_{LT}$ and ${\nu }_{TT}$ are the Poisson ratios that couple the transverse deformation to the axial and transverse deformation when the muscle is stressed in the ${x}_{1}$ and ${x}_{2}$ directions, respectively^25,42^.

In addition to the previous considerations regarding its architecture, the muscle is assumed as a purely elastic material^5,7,11,12,41,43–47^. This hypothesis is often considered in muscle elastographic studies and has been supported by several works that neglect the viscous effects^48,49^. In this way, the longitudinal shear elastic modulus can be calculated through ${V}_{s}$ measured in the fibers' direction (${V}_{s}^{||}$) as:

1 $${\mu }_{L}=\rho {V}_{s}^{||2},$$

where $\rho$ is the muscle density (assumed here to be 1000 kg/m^3^).

Finally, maps of shear elastic modulus (Fig. 1) were calculated at 1.7 Hz with a pixel resolution of 0.1 $\times$ 0.1 mm.

### Protocol

Initially, the volunteers performed two maximal isometric voluntary elbow flexions (each lasting 5 s and resting 120 s between them) with the shoulder and elbow flexed at 90° to determine the maximal voluntary contraction (MVC). The highest MVC value was used to normalize submaximal contractions. Then, volunteers were asked to perform six linear torque ramps (120 s rest between tasks) of isometric elbow flexion from 0 to 30% of MVC over 15 s. In order to correctly execute the torque ramps, they had to follow the path indicated on a monitor placed in front of them. The ${\mu }_{L}$ of BB, BR, and BA muscles was measured twice, in separate trials and random order, during the execution of the tasks. The ultrasonic scanner probe was carefully aligned with respect to the orientation of the muscle fibers. It was placed on the muscle belly, at 70% of the arm’s length distally from the acromion (BB) and 35% of the forearm length distally from the elbow (BR). As in Bouillard et al.^16^ and Hodges et al.^50^, for the deep muscle (i.e., BA), it was placed in the medial and distal part of the arm, near the fold of the joint. To guarantee repeatability concerning the probe locations between trials, these were marked using a waterproof pen.

### Relative longitudinal strain of muscle and ${\upmu }_{\mathrm{L}}$

As is shown in Appendix 1 in Supplementary Material, from the five independent constants needed to describe a transversely isotropic solid such as skeletal muscle, the elasticity in the fiber direction depends only on ${\mu }_{L}$. Likewise, according to the property of the SRS^18–21^ and the acousto-elasticity theory^23–25^, the muscle stress in the fiber direction for a given joint torque ($\tau$) level can be written as:

2 $${\sigma }_{L}=-\frac{1}{{\beta }_{\parallel }}{\Delta \mu }_{L}\left(\tau \right)=-\frac{1}{{\beta }_{\parallel }}\left({\mu }_{L}\left(\tau \right)-{\mu }_{L}\left(0\right)\right),$$

where ${\beta }_{\parallel }$ is a muscle-specific proportionality constant, and ${\mu }_{L}\left(0\right)$ and ${\mu }_{L}\left(\tau \right)$ are the muscle longitudinal shear elastic modulus at rest ($\tau =0$) and contracted according to $\tau$ level, respectively. On the other hand, there is an empirical relationship between Young’s moduli of skeletal muscle in the fiber direction (${E}_{L}$) and its ${\mu }_{L}$, according to which^51^:

3 $${E}_{L}=\gamma {\mu }_{L},$$

where $\gamma$ is another muscle-specific proportionality constant.

From the above, it follows that the relative longitudinal strain of the muscle (${\xi }_{L}$) is a function of ${\mu }_{L}$:

4 $${\xi }_{L}\left(\tau \right)=\frac{{\sigma }_{L}}{{E}_{L}}=-\frac{{\Delta \mu }_{L}\left(\tau \right)}{{\beta }_{\parallel }\gamma {\mu }_{L}\left(\tau \right)}=AC\left(\tau \right),$$

where

5 $$A=-\frac{1}{{\beta }_{\parallel }\gamma },$$

6 $$C\left(\tau \right)=\left(1-\frac{{\mu }_{L}\left(0\right)}{{\mu }_{L}\left(\tau \right)}\right).$$

Specifically, ${\xi }_{L}$ measures the shortening of the muscle in a small portion determined by the spatial resolution of the system, of the order of one ultrasound wavelength (~ 300 μm, according to our experimental setup). As the sarcomeres are aligned in series along the fiber direction, ${\xi }_{L}$ is representative of the longitudinal shortening of the whole muscle as a product of the overlap between the actin and myosin filaments and the increase of ${\mu }_{L}$. In this regard, the coefficient $C\left(\tau \right)\in [0, 1]$ is directly proportional to the ${\xi }_{L}$ and can be calculated from the experimental data of ${\mu }_{L}$.

### Data analysis

For each volunteer, the mean values of ${\mu }_{L}\left(\tau \right)$ in each trial were calculated over a circular region of interest (ROI) of 1 cm in diameter placed in the middle of the elastic field (Fig. 1). These data were synchronized by interpolating with the torque signal to obtain one value for every 1% MVC. Thus, for the three muscles, we calculated the $C\left(\tau \right)$ coefficients (${C}_{BB}\left(\tau \right), {C}_{BR}\left(\tau \right), {C}_{BA}\left(\tau \right)$) in each trial between 0 and 30% MVC, as well as the averaged $\overline{C }\left(\tau \right)$ between the two trials.

### Statistical analysis

To assess the intra-repeatability of the $C\left(\tau \right)$ coefficients obtained in both trials of the isometric flexion ramps, the intraclass correlation coefficient (ICC) was calculated for each muscle in all volunteers. On the other hand, we identified the threshold torque at which the $C\left(\tau \right)$ coefficients differed significantly from $C\left(0\right)$. We performed a repeated-measures ANOVA for each muscle (random factor: participant, between-participant factor: torque) by using PAST 3.21^52^. As in Bouillard et al.^16^, if a main effect was identified for torque (i.e., $C\left(\tau \right)$ changes significantly as torque increases), Duncan’s post-hoc test was applied to detect the first torque value at which the $C\left(\tau \right)$ was statistically different from $C\left(0\right)$ (we will call this point the “lower limit”). On the other hand, the “upper limit” was defined as that point followed by four smaller $C(\tau )$ values. The level of significance was set at *P* < 0.05.

### Ligand-binding analysis

**Figure 2:**
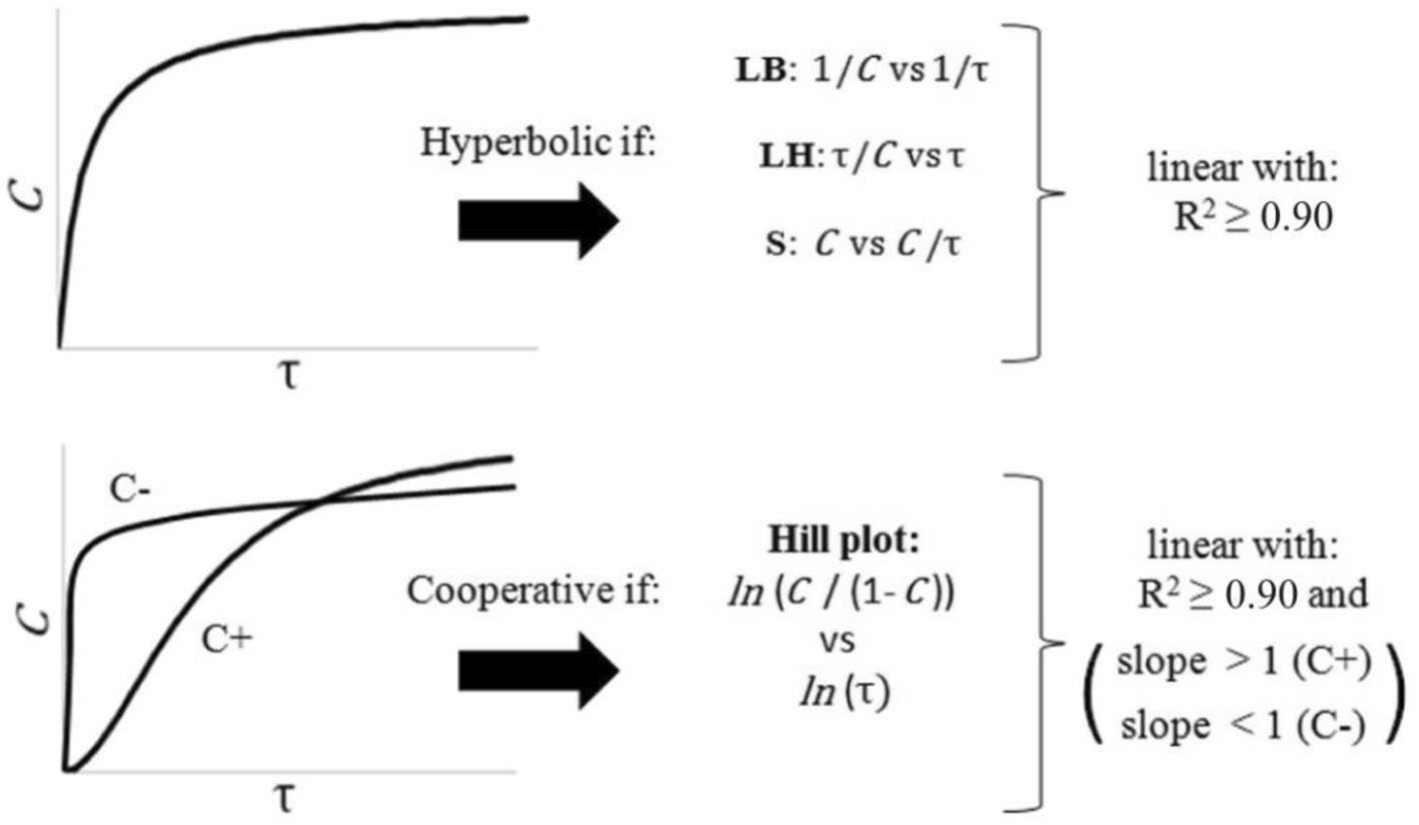
Classical rectification methods of the ligand-binding analysis. These methods were applied for the $C\left(\tau \right)$ coefficients calculated from the ${\mu }_{L}\left(\tau \right)$ values of the BB, BR, and BA muscles.

As $C(\tau )$ is directly related to the fraction saturation of the molecular receptors (Appendix 2 in Supplementary Material), we used conventional characterization methods of receptor-ligand systems to study the dynamical behavior of the elbow flexor muscles^29^ (Fig. 2). If $C$ vs. τ (considered as our “direct plot”) describes a rectangular hyperbolic-like behavior, and the fits of all the following plots are linear with a determination coefficient (R^2^) ≥ 0.90, the binding dynamics will be hyperbolic (H): 1/$C$ vs. 1/τ (Lineweaver–Burk (LB) or double reciprocal plot); τ/$C$ vs. τ (Langmuir–Hanes (LH) plot); $C$ vs. $C$/τ (Schatchard (S) plot). On the other hand, if the direct plot shows a sigmoid curve and the fit of ${\text{ln}}(C/(1-C)$) vs. ${\text{ln}}(\tau )$ (Hill plot) is linear with R^2^ ≥ 0.90, the binding dynamics will be cooperative (positive (C+) if slope > 1, negative (C−) if slope < 1) or non-cooperative (slope = 1). If the Hill plot has a slope less than 1, and the LB, LH, and S plots fit with R^2^ ≥ 0.90, the behavior was classified as a H/C− indeterminacy, which is a common limitation when studying the interaction between receptors and ligands^53^. We perform these analyses in the curve section delimited by the lower limit specified by Duncan’s test and the upper limit. Likewise, if the ICC values denoted a good intra-repeatability of the $C\left(\tau \right)$ coefficients between trials 1 and 2, the previous analyses were performed over the averaged $C\left(\tau \right)$ value of both trials ($\overline{C }\left(\tau \right)$).

### Informed consent

All participants and/or their legal guardians provided their written informed consent to participate in the study and for publication of identifying information/images in an open-access online publication.

## Results

**Figure 3:**
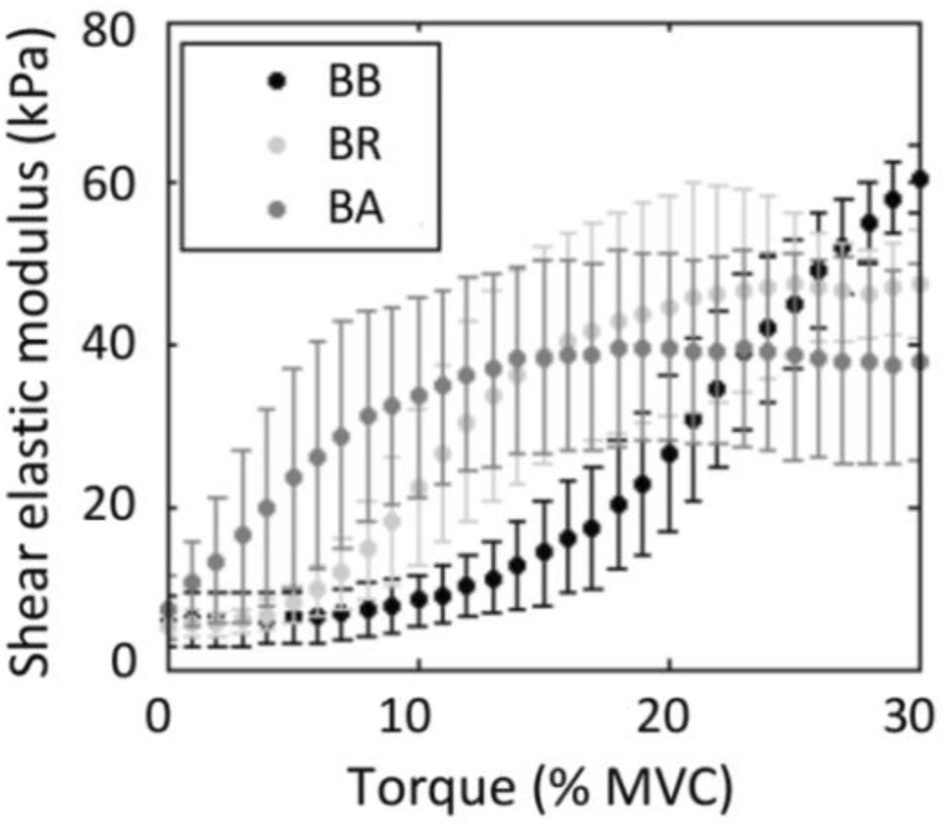
Average values and standard deviation (error bars) of ${\mu }_{L}\left(\tau \right)$ measurements for all volunteers.

**Figure 4:**
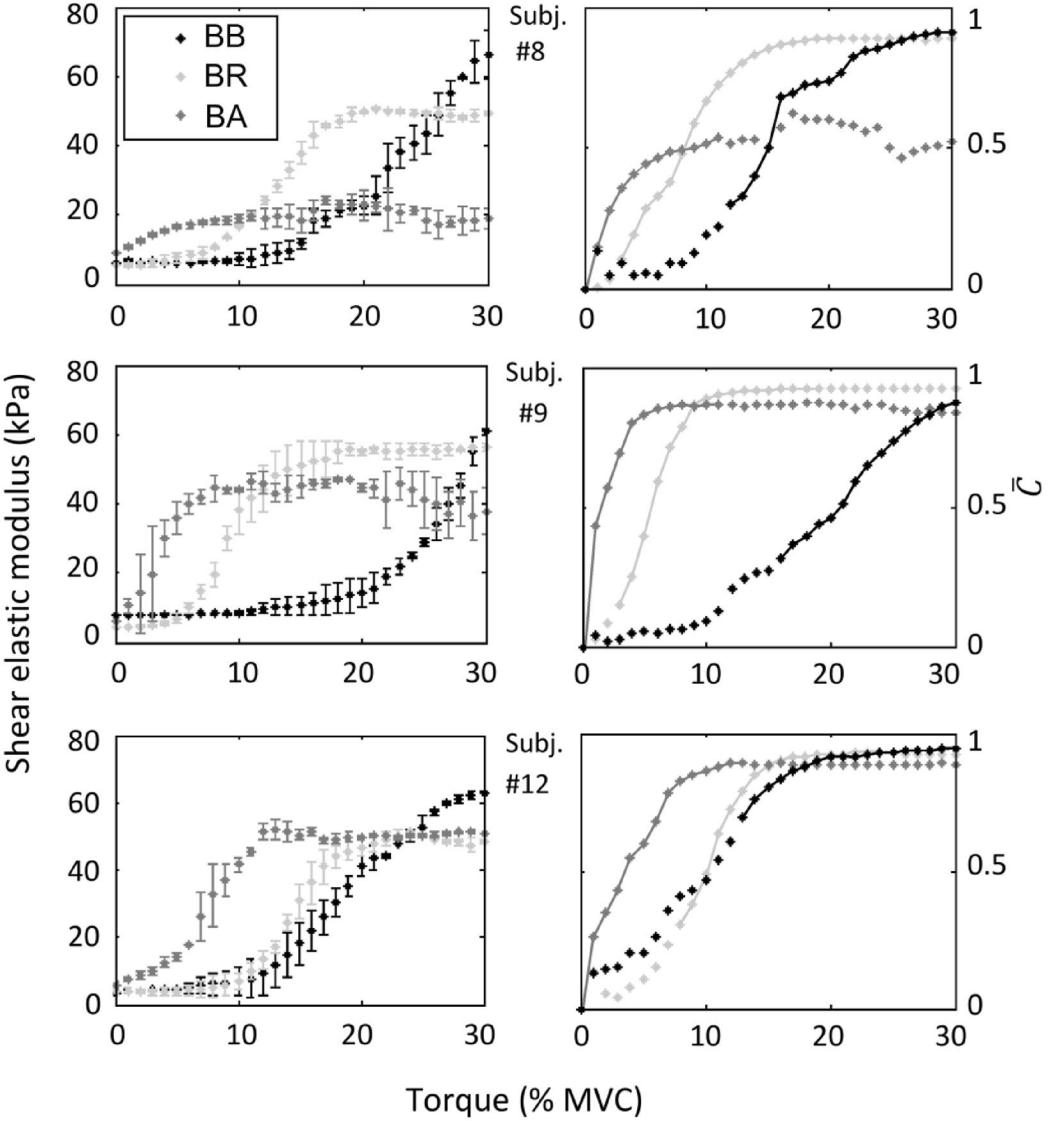
Three individual examples of the change in muscle elasticity regarding the elbow flexion torque for the BB, BR, and BA muscles. The error bar corresponds to the standard deviation of trials 1 and 2. The corresponding $\overline{C }\left(\uptau \right)$ coefficients calculated from the mean value of ${\mu }_{L}\left(\tau \right)$ in trials 1 and 2 are also shown. The segments of the $\overline{C }\left(\uptau \right)$ curves employed for the ligand-binding analysis, delimited by the calculated upper and lower limits, are denoted by solid lines.

From 0 to 30% MVC, the ${\mu }_{L}$ of the muscles displayed different behaviors regarding the elbow flexion torque. This can be observed by averaging the volunteers’ results during the measurements (Fig. 3) and at the individual level (Fig. 4). Although the results showed some individual variability, a common trend was found. Thus, in general terms, the ${\mu }_{L}$ of the BB exhibited a slight or no increase between ~ 0 and 10% MVC, increasing rapidly between ~ 10 and 30% MVC. The BR did not show a significant change in ${\mu }_{L}$ between ~ 0 and 5% MVC, increasing its elasticity eight to nine times between ~ 5 and 20% MVC and remaining around a constant value between ~ 20 and 30% MVC. On the other hand, the BA showed a different behavior regarding the BB and BR. This muscle exhibited the earliest beginning of contraction, increasing moderately its ${\mu }_{L}$ between ~ 0 and 10% MVC, which did not change significantly between ~ 10 and 30% MVC. Table 1 summarizes the above regarding the average behavior of the shear elasticity for the BB, BR, and BA muscles as a function of the elbow flexion torque.

**Table 1:** ${\mu }_{L}$ values and standard deviations (between parentheses) among all volunteers for the BB, BR, and BA muscles during both isometric elbow flexion torque ramps.

	${\mu }_{L}$(kPa)	% MVC						
		0	5	10	15	20	25	30
BB	1st trial	5.78 (3.83)	6.32 (3.78)	9.13 (4.39)	14.38 (8.77)	26.81 (11.55)	44.71 (11.70)	61.07 (4.65)
	2nd trial	5.93 (2.81)	6.48 (2.92)	7.84 (2.70)	14.24 (4.63)	26.82 (9.96)	45.75 (10.32)	60.07 (6.52)
BR	1st trial	5.53 (1.64)	8.91 (3.30)	26.99 (11.09)	43.78 (12.08)	48.39 (9.89)	47.37 (6.38)	47.63 (7.03)
	2nd trial	5.27 (1.54)	7.16 (2.10)	19.98 (7.93)	36.58 (12.40)	44.12 (13.15)	49.28 (11.37)	48.19 (7.28)
BA	1st trial	8.40 (3.89)	24.51 (13.25)	36.22 (11.80)	40.94 (11.51)	43.43 (11.18)	44.11 (12.32)	42.66 (11.91)
	2nd trial	7.42 (3.55)	24.20 (12.48)	35.82 (11.62)	42.14 (14.07)	41.71 (12.96)	38.62 (14.45)	38.84 (14.87)

Concerning the $C\left(\tau \right)$ coefficients calculated from the shear elasticity values between 0 and 30% MVC (Figs. 4, 6A), the ${C}_{BB}\left(\tau \right)$ ranged from 0 to 0.90 ± 0.07 and 0 to 0.90 ± 0.06, while the ${C}_{BR}\left(\tau \right)$ did between 0 to 0.88 ± 0.05 and 0 to 0.88 ± 0.04 (first and second trials, respectively). Meanwhile, the ${C}_{BA}\left(\tau \right)$ varied between 0 to 0.78 ± 0.13 and 0 to 0.78 ± 0.12 (first and second trials, respectively). The high ICC obtained from these values denotes good reproducibility of the ${C}_{BB}\left(\tau \right)$, ${C}_{BR}\left(\tau \right)$, and ${C}_{BA}\left(\tau \right)$ coefficients, calculated from the respective ${\mu }_{L}\left(\tau \right)$ values obtained in both trials for all volunteers (${ICC}_{BB}=$ 0.90 ± 0.08; ${ICC}_{BB}=$ 0.97 ± 0.03; ${ICC}_{BB}=$ 0.89 ± 0.11). In this sense, the averaged $\overline{C }\left(\tau \right)$ value for each muscle was representative of the two isometric flexion ramps. Therefore, in what follows, the analysis is performed based on the ${\overline{C} }_{BB}\left(\tau \right)$, ${\overline{C} }_{BR}\left(\tau \right)$, and ${\overline{C} }_{BA}\left(\tau \right)$ coefficients.

**Figure 5:**
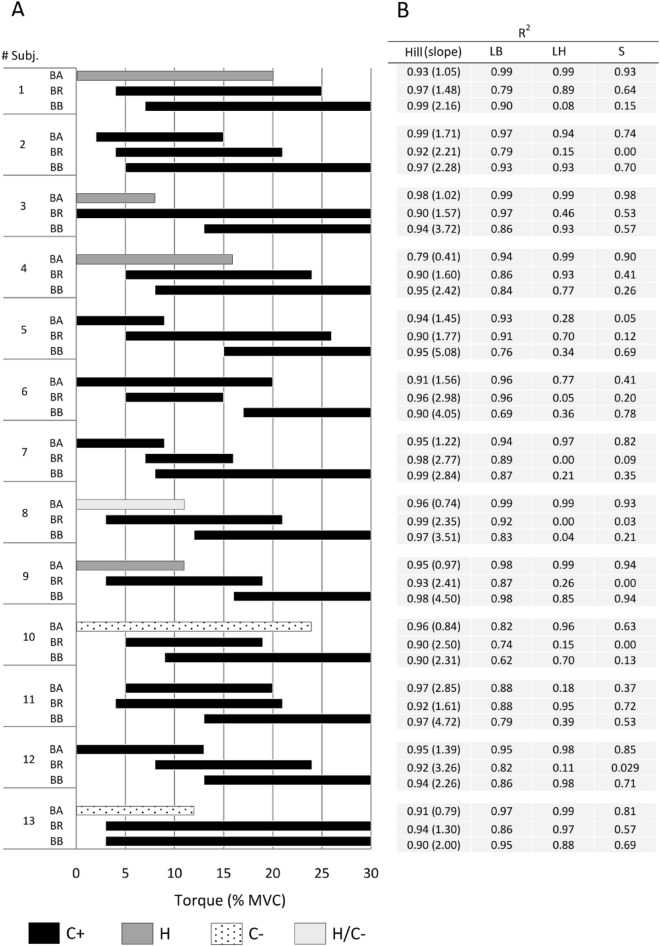
(**A**) Binding dynamics as a function of the elbow flexion torque, resulting from the ligand-binding analysis performed between the lower and upper limits of $\overline{C }\left(\tau \right)$ curves for each muscle. (**B**) R^2^ values resulting from the LB, LH, S, and Hill rectification methods.

The repeated-measures ANOVA showed a significant main effect of the torque level regarding the shear elasticity for BB, BR, and BA muscles (*P* ranged from values < 1.0 × 10^–5^ to 0.026 in all cases). This implies that the ${\mu }_{L}\left(\tau \right)$ was significantly higher as elbow flexion torque increased. In this sense, Duncan’s test set that 11 ± 4%MVC, 4 ± 2%MVC, and 1 ± 1%MVC were the averaged lower limits from which ${\mu }_{L}\left(\tau \right)$ differed significantly from rest, for BB, BR, and BA, respectively. On the other hand, the upper limits were determined at 30 ± 0%MVC (BB), 22 ± 5%MVC (BR), and 14 ± 5%MVC (BA). In this way, the ligand-binding analysis revealed the presence of different contractile dynamics between the lower and upper limits of the ${C}_{BB}\left(\tau \right)$, ${C}_{BR}\left(\tau \right)$, and ${C}_{BA}\left(\tau \right)$ curves of all volunteers. Specifically, the BB and BR muscles show a C+ dynamic, while, depending on the volunteer, the BA muscle can present either a C+, C−, or H dynamic. All of the above is depicted in Fig. 5, as well as the results of the LB, LH, S, and Hill rectification methods that determine such binding behaviors.

## Discussion

### Biomechanical and functional implications of ${\upmu }_{\mathrm{L}}$ and $\mathrm{C}\left(\uptau \right)$ in load sharing

The present work aimed to study the different contractile dynamics exhibited by the elbow flexor muscles during load sharing in isometric conditions. The SW elastography allowed the assessment of such phenomena by measuring the change of the shear elastic modulus of the BB, BR, and BA muscles as the joint torque increased. Thus, we provided additional evidence that supports the work of Bouillard et al.^16^, who showed that at low contraction intensity levels (~ 0–15% MVC), torque is primarily produced by a preferential activity of BA and BR muscles (${\mu }_{L}$ ranged from ~ 10–45 to ~ 3–60 kPa, respectively), while the increase of torque between ~ 10 and 30% MVC is mainly due to BB (${\mu }_{L}$ ranged from ~ 10 to 60 kPa). In this sense, as shown in Table 1 and Figs. 3 and 4, our results concerning the torque-dependent behavior of the shear elasticity of these muscles agreed qualitatively and quantitatively with those of Bouillard et al.^16^ and other previous studies^9,10,12,54^. As a general picture, the anatomical differences could account for these different behaviors among the BB, BR, and BA muscles. For example, at 90° elbow flexion, the BA has approximately half the lever arm of the BB^55–57^. Besides, it has a smaller cross-sectional area and is a uniarticular muscle that inserts next to the elbow joint. These features would be advantageous for developing precise movements at low force levels, thus guaranteeing the first effects of torque generation and joint stability. On the other hand, the BB and BR are biarticular muscles, supplementing the joint torque contribution with slightly higher force levels than BA to additionally stabilize the shoulder and wrist joints. The BB is able to increase the torque to higher levels, helped by their long-moment arm^16^. Compared to BB and BA, the BR has the longest lever arm but the smallest cross-sectional area, thus playing an intermediate role concerning control and torque generation. This muscle is anatomically arranged differently from the BB and BA to provide joint stability by coaptation of the radius in the joint. As the BB, the BR is a biarticular muscle and ensures that the BB does not lift the radius head, expecting a low torque production from it. Thus, it accompanies the BB at the beginning of the contraction, maintaining a constant contribution afterward.

In addition to the inter-muscle anatomical differences, the inter-participant differences regarding the moment arms, cross-sectional areas, and the muscle recruitment thresholds can explain the variability of the shear elasticity vs. torque measurements across the volunteers^16,58^. On the other hand, the different fiber compositions of the muscles must also be considered. The percentage of type I fibers varies, within the 95% confidence limits, from 34 to 51% for BB surface fibers, 40–60% for BB deep fibers, and 30–53% for brachioradialis fibers. Concerning the type II fibers, these proportions are 49–66%, 39–60%, and 47–73%, respectively^59^. These differences in fiber-type distribution between muscles could also explain the different behaviors characterized.

This study provides a new conceptual framework for assessing the load distribution between synergistic muscles. The above, combined with measurements of ${\mu }_{L}\left(\tau \right)$, moment arms, and cross-sectional areas, can be of high interest to precisely study the compensations between individual muscle torques^16^. In this regard, the calculation of $C\left(\tau \right)$ coefficients from the ${\mu }_{L}\left(\tau \right)$ values and the subsequent addition of the ligand-binding framework into the load-sharing analysis revealed the presence of hyperbolic and cooperative behaviors. This finding complements, from a functional point of view, the previous comments regarding the anatomical features that could explain the behavior of each muscle during the load sharing. In this way, our results clearly differentiate the behavior exhibited by the BA from those of the BB and BR. While the BA can display both H, C+, or C− dynamics associated with the torque production at low contraction intensity levels (~ 0–10% MVC), the BB and BR muscles only show C+ dynamics related to the intermediate-high efforts (~ 10–30% MVC) (Fig. 5). As we will discuss later, the causes for which the BA exhibits its particular behavior may reside in peculiarities inherent to its contraction mechanism. Nevertheless, we must also consider the possible incidence of the pennation angle to account for the BA results since the muscle shear elastic modulus decreases as this angle increases Gennisson et al.^8^. This does not have an incidence in the BB muscle as this is a fusiform muscle, nor in the BR since its pennation angle is low (~ 2°) and is minimally affected by the contraction^16,60^. On the contrary, in the BA muscle, the pennation increases by 7.7° from rest to 50% MVC, mostly at contraction intensities below 10% MVC^50^. Thus, as in Bouillard et al.^16^, our measurements of ${\mu }_{L}$ may present some bias at the beginning of the contraction. The above could have influenced the ${C}_{BA}\left(\tau \right)$ calculated within ~ 0–10% MVC, where the H, C+, or C− dynamics appear. Further studies on shear wave propagation in pennate muscles are needed to obtain unbiased results by correcting the incidence of the pennation angle in the shear elastic modulus estimation.

Although it is common practice in muscle elastography to consider only the elastic properties of the muscle, these also exhibit viscoelastic properties. Therefore, the possible incidence of the viscosity in the results should also be discussed. In this respect, Rudenko and Sarvazyan^61^ have shown that the dissipative properties of muscles are determined by the fourth-rank viscosity tensor, which, as the elastic properties, has two independent components. In our experimental protocol, two types of viscous behavior play a role. The first one is a longitudinal viscosity associated with muscle contraction. Previous works performed in the gastrocnemius medialis and the soleus show that longitudinal viscosity effects are relevant in a time scale of ~ 10^–1^ s or lower^62^. Therefore, the longitudinal viscosity effects can be neglected for contraction rates that comprise longer times. This is the case in our experiments since the total ramp time was 15 s, and we sampled the shear elasticity with a frame rate of 1.7 Hz. The second one is the shear viscosity, associated with the shear wave propagation after the “push” of acoustic radiation force. Previous works used Voigt’s linear viscoelastic model to estimate the shear viscosity of biceps brachii from dispersion curves using an SSI device (Gennison et al. 2010). The results show that shear viscosity increases for loaded muscles concerning its rest position, from ~ 1 Pa s at rest to ~ 3.5 Pa for muscles loaded with 4 kg. However, the increase in viscosity does not produce a significant variation in the shear wave speed compared to the value obtained by neglecting the viscosity. In other words, the slope value of the shear wave speed vs. frequency curve is much lower than 1 (~ 1/600 ms^−1^ Hz^−1^). Thus, the results and conclusions reported in our work are not biased by neglecting viscous effects in our model.

**Figure 6:**
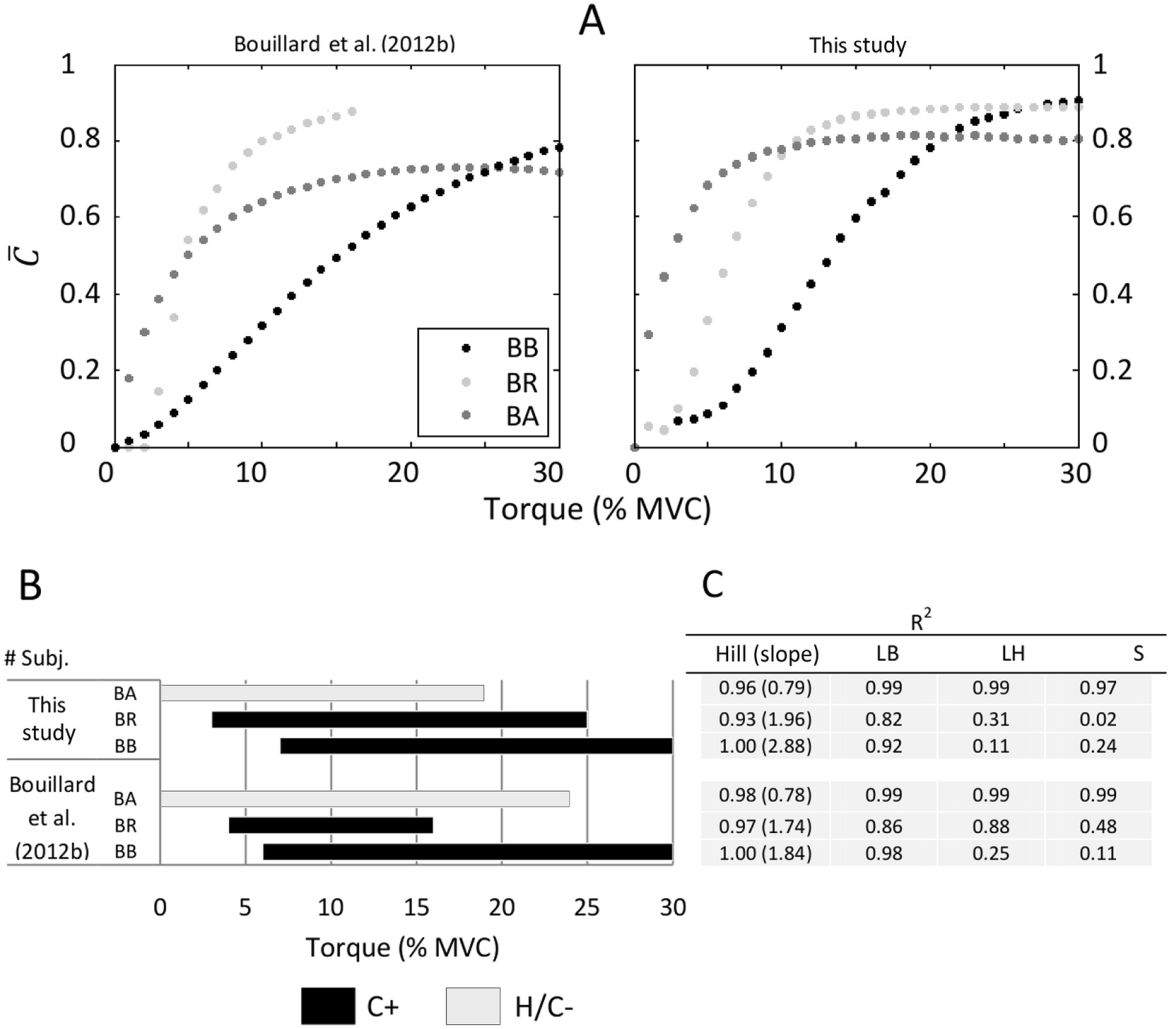
(**A**) Comparison of the $\overline{C }\left(\tau \right)$ coefficients calculated from data of Bouillard et al.^16^ and those of this study (mean values of Fig. 3). It should be noted that in Bouillard et al.^16^, it was not possible to obtain ${\mu }_{L}$ values for BR beyond 16% MVC. (**B**) Ligand-binding behaviors obtained from $\overline{C }\left(\tau \right)$ values in each study. (**C**) R^2^ values resulting from the LB, LH, S, and Hill rectification methods.

Leaving aside the above considerations, it is important to note that the same results are obtained when performing the analysis using the data provided by Bouillard et al.^16^ (Fig. 6). Here, the rate of contraction was slower than our protocol, as the ramps were from 0 to 40%MVC in 30 s. Nevertheless, the ligand-binding analysis for the data of Bouillard et al.^16^ denotes the same dynamical behaviors as our results. This shows the consistency of the elastography-driven approach proposed in the present work to assess the different contractile dynamics that could be behind the muscle synergism of the elbow flexors muscles.

### The biomechanical significance of $\mathrm{C}\left(\uptau \right)$

The present study sheds light on the nature of the coefficients $C\left(\tau \right)$. Equations (4) and (6) state that $C\left(\tau \right)$ coefficients are related to variables changing in the muscle fiber direction. They depend exclusively on the longitudinal shear elastic modulus (${\mu }_{L}$) and are directly proportional to the longitudinal strain of the muscle (${\xi }_{L}$). According to the acousto-elasticity theory, the longitudinal stress in muscle varies linearly with respect to the square of the shear wave velocity in the fiber direction, and thus, with its ${\mu }_{L}$^23–25^. Likewise, the SRS principle states that the longitudinal shortening of the muscle increases its Young’s moduli along the muscle fibers, which depends exclusively on ${\mu }_{L}$ (Refs.^22,51^, Appendix 1 in Supplementary Material). Thus, the SRS principle and the acousto-elasticity theory link ${\xi }_{L}$ and ${\mu }_{L}$ with the shear wave propagation and the contractile processes that determine the shortening in the longitudinal direction of skeletal muscle during the isometric contraction. In this way, the present work provides a novel manner to assess the relative longitudinal deformation of the muscle at increasing levels of isometric force by calculating $C\left(\tau \right)$ exclusively from elastography measurements.

From a functional point of view, the above could also have significance regarding the kinetics of the longitudinal shortening of muscle as the joint torque increases. In particular, it would imply that the length in the fibers’ direction shortens at an increasing, constant, or decreasing rate, depending on whether the dynamics of $C\left(\tau \right)$ is C+, C−, or H, respectively. Thus, our results for the elbow flexors muscles imply that, at low contraction intensity levels (~ 0–15% MVC), the flexion torque is primarily produced by the BA muscle, which displays an H or C− dynamic, thus shortening at a constant or decreasing rate, respectively. On the other hand, the increase in torque after ~ 10–15% of MVC is mainly due to the BB and BR muscles, which follow a C+ dynamic characterized by an increasing rate of shortening. In this sense, these results imply that such shortening dynamics determine the torque-dependent changes in load sharing between the BB, BR, and BA muscles during the isometric flexion of the elbow joint. Besides, they could also account for explaining the non-linear relationship between electromyographic activity vs. torque, in addition to the activation pattern of the motor units classically reported in previous works^12,63,64^.

Related to their direct proportionality concerning ${\xi }_{L}$, our results suggest that $C\left(\tau \right)$ could indicate the amount of the attached actin-myosin cross-bridges as a function of the joint torque level. In this regard, as we show in Appendix 2 in Supplementary Material, such coefficients are closely related to the fraction saturation of molecular receptors (Y ∈ [0, 1]), which is measured by indirect methods (e.g., absorbance measurements) to characterize the ratio of occupied binding sites/total binding sites as the ligand concentration increases^29^. Besides, as discussed below, several previous ligand-binding studies performed on isolated myofilaments have shown the same dynamical behaviors as the $C\left(\tau \right)$ coefficients. Presumably, all the above could imply a link between the micro and macro-scale phenomena associated with muscle contraction, which derives from the SRS principle and the acousto-elasticity theory. This is in good agreement with previous work that has proposed that the SRS principle may provide a measure of the amount of the attached cross-bridges and the contribution of the muscle to joint stiffness^65^. In this respect, it should be noted that this type of micro–macro link has already been observed for other biomechanical properties of skeletal muscle. For example, the force–velocity relation of Hill’s muscle model^66^ can be supported from a molecular perspective. This hyperbolic force–velocity relationship of muscle has been classically regarded as a pure empirical description of the macroscopic force–velocity data^27,67^. However, recent works have established the relationship between the mechanical manifestation in terms of force–velocity data and the kinetics of the cross-bridge cycle driven by ATP hydrolysis, describing how the molecular events within such a process can be transformed into the hyperbolic Hill equation^68,69^.

### Interpretations on the cross-bridges binding dynamics during muscle synergism

The appearance of ligand-binding behaviors, such as hyperbolic and cooperative dynamics, is highly significant. It is important to note that different molecular receptors, for example, hemoglobin and myoglobin, typically exhibit these behaviors. Such molecules have binding sites for their ligands, with specific affinity constants depending on the saturation level and the degree of allosteric modulation. This determines the kinetic behavior of the saturation fraction ($Y$) between the occupied and total binding sites as the ligand concentration increases. The above is analogous to what happens with the actin filament active sites and the myosin heads (subunit S1) during muscle contraction. Here, the actin–troponin–tropomyosin complex (regulated actin) determines muscle contraction and relaxation by mediating the interactions between the myosin heads and the actin filaments^34^. The above depends on specific biochemical factors. Specifically, in absence of Ca^2+^, such a complex causes the muscle to relax by inhibiting the acto-S1 ATPase activity and blocking the binding of S1 to actin. On the other hand, in the presence of Ca^2+^, actin binds to the S1·ADP-Pi complex and activates ATPase, which accelerates phosphate loss and determines muscle contraction^31,33^. Therefore, the actin–troponin–tropomyosin complex regulates the blockade of the actin active sites to myosin heads, thus modulating their binding affinity and the fraction of the myosin heads attached to the actin active sites^70,71^. Since there is a direct relationship between the $Y$ of a molecular receptor and the fraction of the myosin heads attached to the actin active sites, that is, $C\left(\tau \right)$ (Appendix 2 in Supplementary Material), our results suggest that hyperbolic and cooperative behaviors (±) exhibited by the longitudinal shortening of the BB, BR, and BA muscles during their synergistic action, could have its correlate at the molecular level. In this sense, a plausible interpretation for our results is that such ligand-bind dynamics underlie the specific contraction pattern of each muscle, thus determining the intrinsic cross-bridge formation while load sharing as torque increases.

Previous works in solution and isolated muscle fibers could support this interpretation regarding the possible contractile intrinsic mechanisms involved in muscle shortening during synergism. Such studies have shown that receptor–ligand behaviors are manifested in the dynamics of actin–myosin interactions during the reciprocal sliding of the contractile myofilaments^30–40^. For example, Lehrer and Geeves^33^ showed that when the tropomyosin is bound to actin with a stoichiometry of 1 Tm/7 actin subunits, the ATPase activity versus [S1] becomes sigmoid, indicating C+ binding of S1·ATP to actin-tropomyosin. On the other hand, Greene and Eisenberg^30^ studied the binding of myosin heads to the unregulated and regulated actin in the presence of ADP. They found that the S1·ADP complex binds independently (hyperbolic) to the unregulated actin, but it can bind with C+ to regulated F-actin, both in the presence and absence of Ca^2+^. In this respect, the density of attached myosin heads to actin per unit length of thin/thick filament overlap is tightly regulated by the concentration of this ion and by the kinetics of the interactions of the regulatory proteins to the actin^72^. For example, the elastic properties of skeletal muscle reside primarily on the protein titin, which binds to actin in the presence of Ca^2+^^73^. The above suggests a mechanism that can explain the dynamic response of the muscle to active changes in length^74^. Tropomyosin phosphorylation is another molecular mechanism that induces ligand-binding behaviors among contractile filaments in muscle. This was studied by Rao et al.^36^, who carried out a force protocol related to the present study. Specifically, they measured the isometric force/length ratio versus the density of attached myosin heads during a linear force ramp, where the myosin molecules moved reconstituted actin filaments with phosphorylated or dephosphorylated tropomyosin. Their results showed that the actin filament is cooperatively activated by myosin when tropomyosin is phosphorylated. In contrast, when tropomyosin is dephosphorylated, the actin filament behaves hyperbolically. Thus, this work showed that phosphorylation is essential for long-range cooperative activation along the actin filaments. Concerning the C− behavior, Reshetnyak et al.^37^ studied that the binding of the myosin heads to one (state 1) or two (state 2) actin monomers depends on an association constant, which decreases as the myosin heads/actin ratio increases due to the growing steric restrictions. According to these authors, this C− transition from state 1 to state 2 might be associated with force generation and directed movement.

Based on the above, the results of the present work encourage further research to understand how the microscopic processes involved in muscle contraction are manifested at a macro-level in the whole muscle. In this context, we afford a new conceptual and experimental framework to extend the current applications of elastography in muscle biomechanics. Thus, we provide the first results assessing the longitudinal shortening of skeletal muscle through elastography and a ligand-binding approach, suggesting a plausible link to the molecular phenomena underlying muscle functions. On this matter, it should be pointed out that muscle contraction does not depend solely on the characteristics of the cross-bridge binding, so these molecular interpretations could partially explain the characterized muscle mechanical properties. In addition, we are aware that additional evidence from more direct characterization methods is needed to confirm the predictions of the present work regarding the contractile dynamics at the sarcomere level. We believe the microendoscopy could be helpful in this regard^75–77^. Beyond these considerations, the data and methods described here could be the basis to continue delving into the implications of the ligand-binding behaviors in skeletal muscle biomechanics. For example, this work could contribute an advance regarding the estimation of individual muscle forces through elastography, as previous studies have proposed^14,15,22,78^. Our future work will tend to address these challenges mentioned above.

## Conclusions

The present work provides a new framework to assess load sharing during torque production by characterizing the longitudinal deformation of synergistic muscles through measurements of its shear elasticity. In particular, it describes a novel elastography-driven approach to characterize the distinctive role of each synergistic muscle in generating the total joint torque during the isometric flexion of the elbow joint. This approach allowed obtaining the $C\left(\tau \right)$ coefficients from the ${\mu }_{L}$ values of the BB, BR, and BA, which exhibited typical ligand-binding dynamics that could be related to the different functions of each muscle in force generation as torque increases. Specifically, the results of the work suggest that the H, C+, and C− dynamics could be the underlying mechanisms, at the molecular level, of the contractile behavior of each synergistic muscle during the load-sharing. In addition, based on the direct relationship between $C\left(\tau \right)$ and the fraction saturation $Y$, our results also suggest that this coefficient could indicate the amount of cross-bridges attached as a function of torque. Therefore, this work extends the applications of elastography by showing its possible utility in inferring contractile processes at different scales that determine the biomechanical properties of the whole skeletal muscle.

### Supplementary Information

Supplementary Tables.

Supplementary Information.

## Data Availability

The data that support the findings of this study are available from the corresponding author upon reasonable request from a qualified researcher. The Appendix 1 and 2 can be found in the Supplementary Information. The complete dataset of the present work is available in the Supplementary Tables.
