# Supplementary material for: Load sharing between synergistic muscles characterized by a ligand-binding approach and elastography
Source: Sci Rep. 2023 Oct 25;13:18267. doi: 10.1038/s41598-023-45037-y (PMC10600237; doi:10.1038/s41598-023-45037-y)
Supplement: Supplementary file 2 — Supplementary Information. [file 41598_2023_45037_MOESM2_ESM.pdf]

Load sharing between synergistic muscles characterized by a ligand-binding approach and elastography

Gustavo A. Grinspan, Liliam Fernandes de Oliveira, Maria Clara Brandao, Andrés Pomi, Nicolás Benech

Appendix 1: Detailed deduction of the relationship between ξ_L and μ_L.

Skeletal muscle can be considered as a transversely isotropic (TI) material (Gennisson et al., 2003). These types of material are characterized by an axis of symmetry such that the material properties are unchanged by rotation about this axis or reflections about any plane parallel to it. In the case of skeletal muscle, the symmetry axis is defined by the direction of the muscle fibers.

Assuming small displacements and a linear stress-strain relationship, the generalized Hooke's law can be used to relate the stress tensor (τ) with the strain tensor (ϵ) via:

$$\tau_{ij} = C_{ijkl}\,\epsilon_{kl} \qquad (\text{A}.1.1)$$

where the sum over repeated indices is understood. In the general case, five independent constants are needed to describe the elastic tensor in a TI material. Using Voigt notation, the elastic tensor $\boldsymbol{C}$ is given by:

$$\boldsymbol{C} = \begin{pmatrix} c_{11} & c_{11}-2c_{66} & c_{13} & 0 & 0 & 0 \\ c_{11}-2c_{66} & c_{11} & c_{13} & 0 & 0 & 0 \\ c_{13} & c_{13} & c_{33} & 0 & 0 & 0 \\ 0 & 0 & 0 & c_{44} & 0 & 0 \\ 0 & 0 & 0 & 0 & c_{44} & 0 \\ 0 & 0 & 0 & 0 & 0 & c_{66} \end{pmatrix} \qquad (\text{A}.1.2)$$

where x_3 is the symmetry axis. The relationship between τ and ϵ can also be expressed in terms of the compliance tensor $\boldsymbol{S} = \boldsymbol{C}^{-1}$ via $\epsilon_{ij} = S_{ijkl}\,\tau_{kl}$. The compliance tensor S can be expressed in terms of the elastic constants $c_{\alpha\beta}$ given in equation 2, but it is customary to express it in terms of the engineering constants, Young's modulus (E), Poisson's modulus (ν) and shear modulus (μ) as O'Donnel & Skovoroda (2004):

$$\boldsymbol{S} = \begin{pmatrix} 1/E_T & -\nu_{TL}/E_T & -\nu_{LT}/E_L & 0 & 0 & 0 \\ -\nu_{TL}/E_T & 1/E_T & -\nu_{LT}/E_L & 0 & 0 & 0 \\ -\nu_{LT}/E_L & -\nu_{LT}/E_L & 1/E_L & 0 & 0 & 0 \\ 0 & 0 & 0 & 1/\mu_L & 0 & 0 \\ 0 & 0 & 0 & 0 & 1/\mu_L & 0 \\ 0 & 0 & 0 & 0 & 0 & 1/\mu_T \end{pmatrix} \qquad (A.1.3)$$

where L stands for longitudinal (along the muscle fibers) and T for transverse (perpendicular to the fibers). The five independent constants are reduced to three if the skeletal muscle is considered incompressible because, in this case, $\nu_{LT} = 1/2$ and $\nu_{TL} = 1 - E_T/2E_L$ (Rouze et al., 2021). Thus, we can choose any three independent from the tensor $\boldsymbol{S}$, say E_L , μ_L and μ_T.

The longitudinal and transversal shear moduli μ_L and μ_T can be assessed experimentally by shear wave elastography (SWE) measurements. This method uses several “push” beams to generate plane shear wave propagation within the muscle. Then, a speckle tracking technique is used to follow the shear wave as it propagates while time of flight measurements allow us to estimate its speed v (Bercoff et al., 2004). The polarization of the shear wave (i.e. the push direction) is always perpendicular to the muscle fibers. The transducer can be oriented along or perpendicular to the fibers, which allows assessing v_L or v_T, respectively. Finally, the shear wave moduli can be computed from the speed values as:

$$\mu_j = \rho v_j^2 \qquad (A.1.4)$$

where j is either L or T depending on the orientation of the transducer with respect to the fibers and ρ is the material density of the muscle which is assumed to be that of water ρ = 1000 kg/m^3.

The SWE method can be used to assess the shear wave speed v during an isometric contraction of the muscle. When in contraction, the muscle is subject to a normal stress $\tau_{33} = \sigma_L$ along the fibers and thus, v becomes a function of σ_L. The relationship between v and σ_L can be found on the basis of an acousto-elastic theory for TI materials developing the elastic strain energy to third order in terms of the strain tensor (Remeniéras et al., 2021). The result shows that it is possible to write the acousto-elastic equation for the shear wave speed as:

$$\rho_0 v_L^2 = \mu_L(0) - \beta_{\parallel}\sigma_L(\tau) \qquad (A.1.5)$$

for the shear wave propagating parallel to the fibers and

$$\rho_0 v_T^2 = \mu_T(0) + \beta_\perp \sigma_L(\tau) \qquad (A.1.6)$$

for the one propagating perpendicular to the fibers, where τ is the external torque and $\mu_{L(T)}(0)$ is the value of the shear modulus at rest (0 external torque). The coupling constants $\beta_\parallel$ and $\beta_\perp$ depend on the second and third-order parameters of the strain energy. Previous works have shown empirically that while v_L changes significantly during an isometric contraction, v_T has little or no variation (Remeniéras et al., 2021). The experiments show that $\beta_\parallel$ is negative, indicating an increase of v_L with tension. On the other hand, $\beta_\perp$is negative and one or two orders of magnitude lower than $\beta_\parallel$, indicating a small softening in the transverse direction with tension. Therefore, for the purposes of this work, we will not consider μ_T as a relevant variable during the contractions and will not include its measurement in the experimental protocol.

As far as E_L is concerned, it is expected to have a strong correlation with v_L. As the muscle contracts, it becomes more difficult to achieve a longitudinal deformation for a given stress value. Therefore, an increase in E_L during contraction is expected, as happens with v_L, since the muscle becomes stiffer. Although it is not possible to obtain this relationship theoretically, experimental works have shown a linear relationship between them (Eby et al., 2013). Therefore, in this work, we will assume this behavior writing:

$$E_L(\tau) = \gamma \rho_0 v_L(\tau)^2 = \gamma \mu_L(\tau) \qquad (A.1.7)$$

Thus, of the three independent constants needed to describe the elastic behavior of the muscle, only μ_L is relevant during an isometric contraction.

Muscle contraction is carried out by shortening the muscle fibers, which produces a longitudinal instantaneous strain $\epsilon_{33} = \xi_L$. If the normal stresses perpendicular to the fibers (τ_{11} and τ_{22}) are zero or negligible compared to σ_L, we can write:

$$\xi_L(\tau) = \frac{\sigma_L(\tau)}{E_L(\tau)} \qquad (A.1.8)$$

Using equations (A 1.5) and (A 1.7), the longitudinal strain can be written as a function of μ_L as:

$$\xi_L = -\frac{1}{\beta_\parallel}\frac{\mu_L(\tau) - \mu_L(0)}{\gamma \mu_L(\tau)} = A\frac{\Delta\mu_L}{\mu_L} \qquad (A.1.9)$$

where $A = -1/\beta_{\|}\gamma$ and $\Delta\mu_L = \mu_L(\tau) - \mu_L(0)$.

Appendix 2: Relationship between the fraction saturation of a molecular receptor and $C(\tau)$.

The following reaction represents the simplest ligand (L) binding scheme for an unsaturated molecular receptor (R):

$$R + L \rightleftharpoons \text{RL} \qquad (\text{A}.2.1)$$

where RL is the receptor-ligand complex. The fraction saturation (Y) of such a receptor is defined as the ratio between their occupied (saturated) and the total binding sites, which can be expressed in terms of the concentration of the reactants and products of Eq. (A.2.1) (Wyman et al., 1990):

$$Y = \frac{occupied\ sites}{total\ sites} = \frac{[RL]}{[R] + [RL]} \qquad (\text{A}.2.2)$$

This fraction is commonly measured by indirect methods. For example, the fraction saturation of the RL complex could be characterized by UV light absorbance measurements. Here, it is possible to obtain the fraction saturation of RL by measuring its light absorbance and comparing it with respect to a reference value (for example, the light absorbance of R).

Skeletal muscle can be considered analogous to a receptor-ligand system, as its contraction depends on the formation of cross-bridges by increasing the fraction of the myosin heads attached to the actin active sites. As shown in previous studies, the stiffness of the cross-bridge array at the sarcomere level is linearly related to the number of attached cross-bridges (Blange et al., 1972; Morgan et al., 1977). Thus, according to the short-range stiffness principle (SRS), the axial shortening of the muscle during its isometric contraction increases its longitudinal shear elastic modulus (μ_L), being proportional to the attached cross-bridges (Zonnino et al., 2019). In addition, Van Eesbeek et al. (2010) have proposed that the SRS may indicate the amount of attached cross-bridges and the muscle contribution to joint stiffness.

Since μ_L is proportional to the amount of active cross-bridges, similar to the light absorbance measurements, the elastography could characterize the fraction of the attached actin-myosin cross-bridges as a function of the joint torque level. Thus, the $\mu_L(0)$ value implies that the sarcomere has no or a basal proportion of attached cross-

bridges, that is, $\mu_L(0) \sim [R]$. On the other hand, when the muscle contracts, a certain amount of cross-bridges are formed according to the torque level, and its μ_L varies with respect to the basal (resting) state such that $\Delta\mu_L(\tau) = \mu_L(\tau) - \mu_L(0) \sim [RL]$. In this way, it is possible to express Y in terms of μ_L:

$$Y = \frac{[RL]}{[R] + [RL]} \approx \frac{\mu_L(\tau) - \mu_L(0)}{\mu_L(0) + (\mu_L(\tau) - \mu_L(0))} = \frac{\Delta\mu_L(\tau)}{\mu_L(\tau)} = C(\tau) \qquad (\text{A}.2.3)$$

Therefore, the above shows that the fraction saturation of a molecular receptor corresponds to the $C(\tau)$ coefficient in the skeletal muscle, which can be characterized by elastography.
